# Comprehensive profiling of serotypes, antimicrobial resistance and virulence of *Salmonella* isolates from food animals in China, 2015–2021

**DOI:** 10.3389/fmicb.2023.1133241

**Published:** 2023-04-04

**Authors:** Lili Guo, Tianan Xiao, Liqin Wu, Yan Li, Xiaoxiao Duan, Wenhua Liu, Kaidi Liu, Wenjie Jin, Hao Ren, Jian Sun, Yahong Liu, Xiaoping Liao, Yongda Zhao

**Affiliations:** ^1^College of Veterinary Medicine, Qingdao Agricultural University, Qingdao, China; ^2^Qingdao Bolin Biotechnology Co., Qingdao, China; ^3^Guangdong Veterinary Medicine and Feed Supervision Institute, Guangzhou, China; ^4^Qingdao Municipal Center for Animal Disease Control and Prevention, Qingdao, China; ^5^Laboratory of Veterinary Pharmacology, College of Veterinary Medicine, South China Agricultural University, Guangzhou, China

**Keywords:** *Salmonella*, resistance phenotypes, biofilm formation, resistance genes, virulence genes

## Abstract

**Introduction:**

*Salmonella* is a ubiquitous foodborne pathogen and mainly transmitted to human farm-to-fork chain through contaminated foods of animal origin.

**Methods:**

In this study, we investigated the serotypes, antimicrobial resistance and virulence of *Salmonella* from China.

**Results:**

A total of 617 *Salmonella* isolates were collected from 4 major food animal species across 23 provi nces in China from 2015-2021. Highest *Salmonella* prevalence were observed in Guangdong (44.4%) and Sandong (23.7%). Chickens (43.0%) was shown to be the major source of *Salmonella* contamination, followed by pigs (34.5%) and ducks (18.5%). The number of *Salmonella* increased significantly from 5.51% to 27.23% during 2015–2020. *S*. Derby (17.3%), *S*. Enteritidis (13.1%) and *S*. Typhimurium (11.4%) were the most common serotypes among 41 serotypes identifiedin this study. Antibiotic susceptibility testing showing that the majority of the *Salmonella* isolates were resistant to neomycin (99.7%), tetracycline (98.1%), ampicillin (97.4%), sulfadiazine/trimethoprim (97.1%), nalidixic acid (89.1%), doxycycline (83.1%), ceftria xone (70.3%), spectinomycin (67.7%), florfenicol (60.0%), cefotaxime (52.0%) and lomefloxacin (59.8%). The rates of resistance to multiple antibiotics in *S*. Derby and *S*.Typhimurium were higher than that in *S*. Enteritidis. However, the rate of resistance to fosfomycin were observed from higher to lower by *S*. Derby, *S*. Enteritidis, and *S*. Typhimurium. Biofilm formation ability analysis found that 88.49%of the Salmonella were able to produce biofilms, of which 236 Salmonella isolates were strong biofilm producer. Among the 26 types of antibiotics resistance genes (ARGs) were identified in this study, 4 ARGs (tetB,sul2,aadA2, and aph(3’)-IIa) were highly prevalent. In addition, 5 β-lactam resistance genes (*bla*_TEM_, *bla*_SHV_, *bla*_CMY-2_, *bla*_CTX-M_, and *bla*_OXA_) and 7 quinolone resistance genes (*oqxA*, *oqxB*, *qnrB*, *qnrC*, *qnrD*, *qnrS*, and *qeqA*) were detected among these isolates. 12 out of 17 virulence genes selected in this study were commonly presented in the chromosomes of tested isolate, with a detection rate of over 80%, including *misL*, *spiA*, *stn*, *pagC*, *iroN*, *fim*, *msgA*, *sopB*, *prgH*, *sitC*, *ttrC*, *spaN*.

**Discussion:**

This study provided a systematical updating on surveillance on prevalence of *Salmonella* from food animals in China, shedding the light on continued vigilance for *Salmonella* in food animals.

## 1. Introduction

*Salmonella*, a foodborne pathogen, causes gastroenteritis with severity ranging from diarrheal symptoms to death (Bae et al., 2013). *Salmonella* is one of the most common pathogens causing foodborne poisoning and is listed as a significant cause of illness and death by the World Health Organization (Chang et al., 2022). To date, approximately 2,600 serotypes have been identified and the majority of them can cause cross-infection between animals and humans (Raufu et al., 2021). Among these, *Salmonella* Enteritidis (*S*. Enteritidis) and *Salmonella* Typhimurium (*S*. Typhimurium) are the frequently isolated as the most common serovars worldwide (Monte et al., 2021; Raufu et al., 2021). Some studies indicated that *Salmonella* can be transmitted to humans along the farm-to-fork continuum, commonly through contaminated foods of animal origin, namely poultry, swine, pigeon, cattle, fish etc. (Jajere, 2019; Stevens and Kingsley, 2021). Over the last few years, periodic outbreaks of *Salmonella* have been reported worldwide to result in tremendous economic losses.

Antibiotic therapy acts as the first-line approach to treat and control *Salmonella* infection, and antibiotic-resistance in *Salmonella* has received global attention (Vinueza-Burgos et al., 2019). Cephalosporins and fluoroquinolones are the conventionally used for the treatment against *Salmonella* infection. Unfortunately, the emergence of extended-spectrum β-lactamase (ESBLs) producers and fluoroquinolone resistant strains among *Salmonella* posed a challenge for clinical treatment of *Salmonella* infection by dampening the antibiotic efficacy (Mąka and Popowska, 2016). The major ESBLs families are found as the *bla*_TEM_, *bla*_SHV_, and *bla*_CTX-M_ clinically, of which, the *bla*_CTX-M_ group presented as most commonly identified ESBL type in *Salmonella* spp. (Bai et al., 2016). The plasmid-mediated quinolone resistance (PMQR) involves acquisition of (i) *qnr* genes (*qnrA*, *qnrB*, *qnrS*, *qnrC*, *qnrD*), (ii) the *aac(6^，^)-lb-cr* gene, and (iii) *oqxAB* and *qepA* genes (Robicsek et al., 2006; Correia et al., 2017). They predominantly contributed spreading of fluoroquinolone resistance *Salmonella* spp. at human–animal interface (Cuypers et al., 2018). And it is even worse after irrational usage of antibiotics in agriculture for decades, leading to further development of resistances to diverse antibiotics in *Salmonella* (Cuypers et al., 2018). These multidrug resistant (MDR) *Salmonella* now become a life-threatening concern to public health with increased morbidity and mortality (Economou and Gousia, 2015; Bai et al., 2016). With growing awareness to tackle the further development of antibiotic resistance in *Salmonella*, longitudinal surveillance programs have been launched by governments or researchers, providing valuable epidemiological data for risk assessment and medication guidance.

As mentioned above, colonized *Salmonella* exert harmful impact on host health. The pathogenicity is generally dependent upon their virulence which responsible for bacterial adhesion, invasion and replication within the host, thereafter damage infected tissues. The virulence can be encoded by genes presented either on the bacterial chromosome or plasmids, to work symmetrically to infect the hosts (Raufu et al., 2021). To date, 24 SPIs have been identified and characterized, which are involved in different stages of *Salmonella* infection. Of which, SPIs-1-5 were common to all serotypes of *Salmonella*. SPI-1 and SPI-2 contain a large number of virulence genes associated with the intracellular pathogenesis and co-encode T3SS, a molecular syringe (Wang et al., 2020; Sedrakyan et al., 2022). Other SPIs are present to varying degrees in *S.* Enteritidis subspecies, some encoding other secretion systems such as T1SS and T6SS, other effector molecules, and fimbriae (Sedrakyan et al., 2022). Among the virulence associated genes, different virulence factors played different role in pathogenesis. Fimbriae virulence genes (*sefA*, *lpfA*, *lpfC*, *csgA*, and *pefA*) promote bacterial binding to intestinal epithelium (Webber et al., 2019). Fimbriae are essential for the synthesis of extracellular polymeric substances (EPS), which are involved in organism formation and environmental persistence. *Spv*B associated with the *Salmonella* virulence plasmid, responsible for intracellular maintenance and bacterial survival (Kong-Ngoen et al., 2022). The *invA*, *orgA*, *sipB*, *prgH*, and *spaN* genes are related to the structure of TTSS (Type Three Secretion System) (Khajanchi and Foley, 2022). *SifA*, *avrA*, *sopE*, *sopB* and *sivH* genes associated to *Salmonellosis* outbreaks (Webber et al., 2019). *CsgA* is associated with pathogenic mechanisms and autoagglutination, promoting inflammation and increasing invasion.

Considering the consumption of animal-derived food accumulatively increase for decades, the prevalence and characterization of *Salmonella* in contaminated food of animal source is of interest. However, the previous works majorly focused on certain provinces or territories, rarely reaching to the in-depth understating towards evolutionary trajectories and transmission dynamics of *Salmonella* nationwide (Li et al., 2013; Zhu et al., 2019; Chen et al., 2020). Therefore, we investigated the distribution characteristics, phenotypic and genotypic antimicrobial resistance and virulence profiling of *Salmonella* isolates from 4 major food animals in China.

## 2. Materials and methods

### 2.1. *Salmonella* isolation and identification

The prevalence of *Salmonella* in food animals (chickens, ducks, goose and pigs) was monitored by volunteers from 23 provinces in China from 2015 to 2021. A total of 2,127 suspected samples [(heart, *n* = 126), (liver, *n* = 806), spleen (*n* = 101), intestine (*n* = 569), stool (*n* = 107), and buccal swabs (*n* = 418)] were collected from food animals. Then, they were sent to the national risk assessment laboratory for antimicrobial resistance of animal original bacteria in South China Agricultural University in Guangdong, China, to isolate and identify the *Salmonella*. The isolation and identification of *Salmonella* strains were performed according to the Standard ISO-6579 (International Organization for Standardization, 2002) protocol (Assaf et al., 2020). The suspected isolates were incubated in LB broth in a constant temperature shaker at 37°C for 12 ~ 16 h. The inoculation loop was picked to inoculate into *Salmonella* chromogenic medium and incubated at 37°C for 12 ~ 16 h. The red single colonies were picked and inoculated in LB broth medium and incubated at 37°C for 16 ~ 18 h. Isolates with typical *Salmonella* phenotypes were further confirmed using API 20E test strips (bioMerieux, Marcy-l’Etoile, France). All confirmed *Salmonella* isolates were serotyped according to the White-Kauffmann-Le Minor scheme using *Salmonella* diagnostic antisera kit.

### 2.2. Antimicrobial susceptibility testing

Antimicrobial susceptibility testing was performed using the agar dilution method. The susceptibility of *Salmonella* isolates were tested to the 23 antimicrobial agents including ampicillin (AMP, 86.0%), cefotaxime (CTX, 89.3%), ceftriaxone (CRO, 89.0%), aztreonam (ATM, 97.6%), amikacin (AMK, 711.2 U/mg), gentamicin (GEN, 633 U/mg), spectinomycin (SPT, 98.8%), neomycin (NEO, 97.5%), ciprofloxacin (CIP, 84.9%), enrofloxacin (ENR, 99.5%), norfloxacin (NOR, 96.7%), levofloxacin (LEV, 97.6%), lomefloxacin (LOM, 99.9%), gatifloxacin (GAT, 95.2%), nalidixic acid (NAL, 90.0%), tetracycline (TET, 99.5%), doxycycline (DOX, 97.6%), florfenicol (FFC, 99.5%), azithromycin (AZM, 98%), Fosfomycin (FOS, 95.0%), colistin (CS, 90.0%), sulfadiazine/trimethoprim (S/T, 99.5%/99.4%), and meropenem (MEM, 99.0%). All antibiotics were purchased from Beijing Solebro Technology Co. *E. coli* strain ATCC 25922 was used as the quality control strain. MICs were interpreted by referring to standards from CLSI documents M100-S28. The resistant breakpoints used were as follows: ≥32 μg/l for AMP, ≥4 μg/l for CTX, ≥4 μg/l for CRO, ≥16 μg/l for ATM, ≥64 μg/l for AMK, ≥16 μg/l for GEN, ≥128 μg/l for SPT, ≥16 μg/l for NEO, ≥4 μg/l for CIP, ≥2 μg/l for ENR, ≥16 μg/l for NOR, ≥8 μg/l for LEV, ≥8 μg/l for LOM, ≥8 μg/l for GAT, ≥32 μg/l for NAL, ≥16 μg/l for TET, ≥16 μg/l for DOX, ≥16 μg/l for FFC, ≥32 μg/l for AZM, ≥256 μg/l for FOS, ≥8 μg/l for CS, ≥16 μg/l for S/T, and 4 μg/l for MEM. The strain resistant to at least one antibiotic agent from three or more antibacterial categories is known as MDR (Gnimatin et al., 2022).

### 2.3. Biofilm formation

The assay of biofilm formation was performed with previous reference (Gomez-Baltazar et al., 2019). The isolates were incubated in 96-well sterile microplates at 28°C for 48 h. Then the non-adherent bacteria were removed from the 96-well microplates. The samples were then anhydrous methanol-fixed and crystal violet staining for biofilm, washed by PBS. The absorbance at 595 nm was determined using a plate reader after dissolving by glacial acetic acid. The above operation was technically repeated for 3 times, and the results was presented as the mean of the 3 biological replicates.

### 2.4. Detection of resistance genes and virulence genes

Thirty-two antibiotic resistance genes (ARGs) and seventeen virulence genes were examined in all isolates. First, DNA was extracted using the standard boiling method (Guo et al., 2022). Then, the target genes were amplified using polymerase chain reaction (PCR) and the sequence of primers, as well as the size of amplicons and the corresponding reference of each gene are presented in Supplementary Tables S1, S2 (Skyberg et al., 2006; Sánchez-Jiménez et al., 2010; Akinyemi et al., 2011; Parvathi et al., 2011; Maravić et al., 2013; Fang et al., 2019; Sun et al., 2019; Chen et al., 2020; Han et al., 2020). Ten randomly selected PCR products were sequenced per gene, then the sequence alignment analysis with NCBI database was carried out to validate the accuracy of the sequences.

### 2.5. Statistical analysis

The *χ*^2^ test and Fisher’s exact test were used to perform the statistical analysis. For all models, we considered *p* < 0.05 as statistical significance and then performed 2-sided probability on those results by using SPSS version 23.0 (IBM, Chicago, IL, United States). Correlation (*r*) was analyzed using SPSS version 23.0 (IBM, Chicago, IL, United States).

## 3. Results

### 3.1. Prevalence and serotypes of *Salmonella*

**Figure 1:**
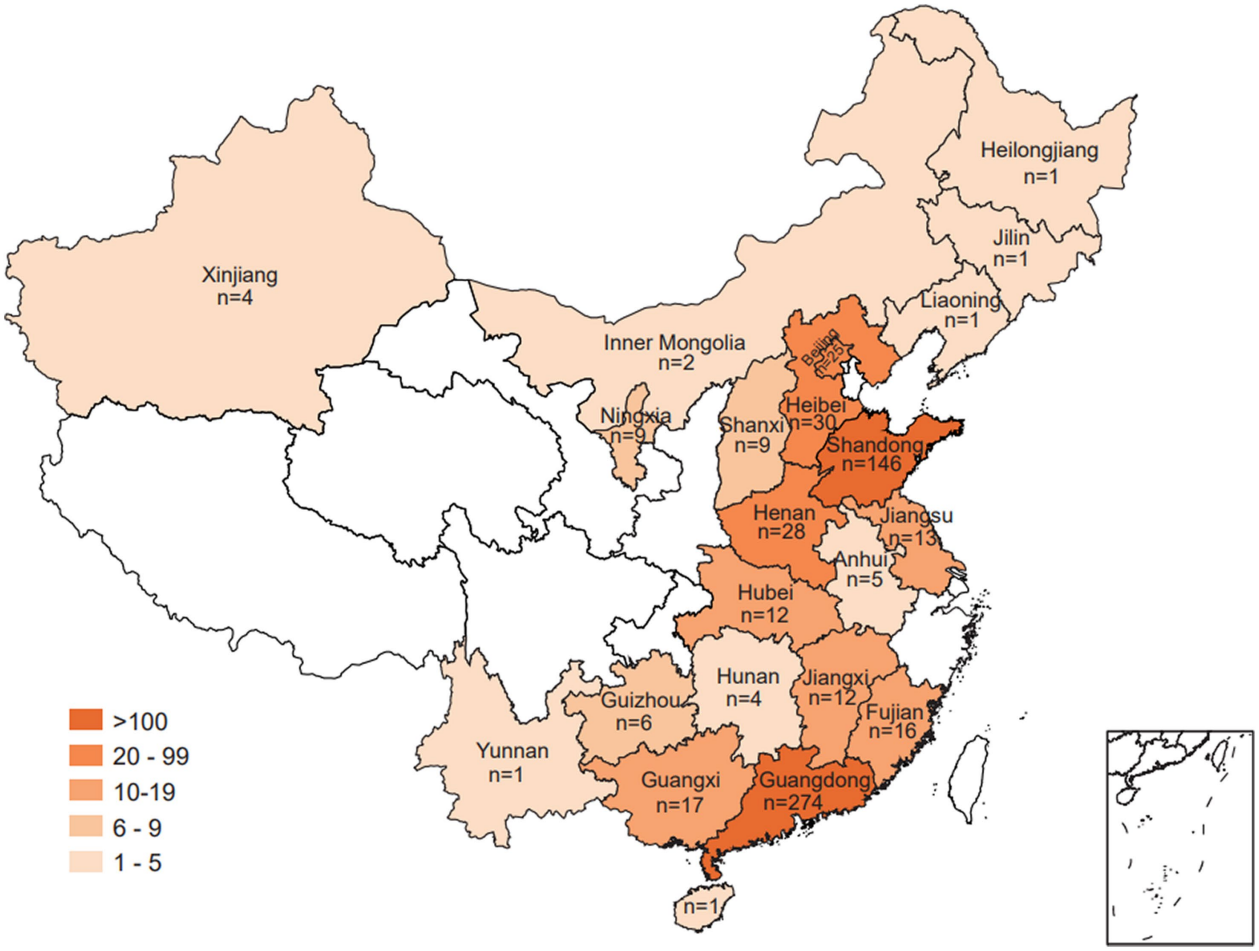
Geographic presentation of *Salmonella* isolates collected from food animals across different provinces of China.

**Figure 2:**
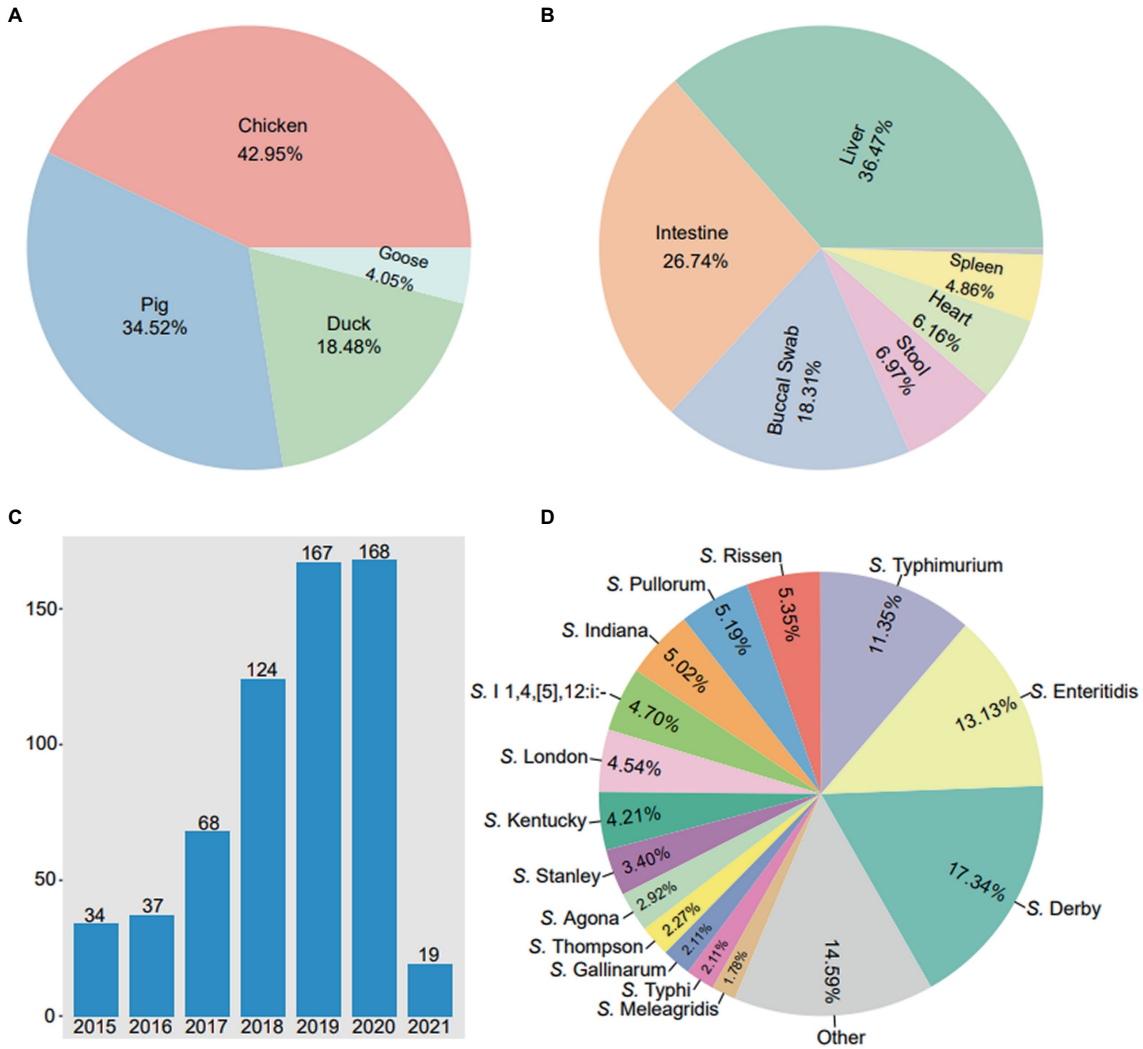
The prevalence of 617 *Salmonella* isolates from food animals in China. **(A)** The detection rates of *Salmonella* isolates in diffierent hosts. **(B)** The detection rates of *Salmonella* isolates in different body sites. **(C)** The annual number of *Salmonella* isolates in 2015 ~ 2021. **(D)** The detection rates of various serovars in 617 *Salmonella* isolates.

In this study, a total of 617 *Salmonella* isolates were obtained from food animals across 23 provinces in China. Notably, the majority of *Salmonella* isolates were recovered in Guangdong (44.41%, 274/617) and Shandong (23.66%, 146/617) (Figure 1). Among 617 *Salmonella* isolates, 265 (42.95%) were isolated from chickens, 213 (34.52%) from pigs, 114 (18.48%) from ducks, and 25 (4.05%) from geese (Figure 2A). The *Salmonella* isolates found to be likely enriched in liver (225/617, 36.47%), followed by in intestine (165/617, 26.74%), buccal swabs (113/617, 18.31%), stool (43/617, 6.97%), heart (38/617, 6.16%), and spleen (30/617, 4.86%) (Figure 2B). The time-resolved prevalence of *Salmonella* isolates is dynamically increasing from 5.51% (34/617) in 2015, 6.00% (37/617) in 2016, 11.02% (68/617) in 2017, 20.10% (124/617) in 2018, 27.07% (167/617) in 2019 to 27.23% (168/617) in 2020 (Figure 2C). The *χ*^2^ test revealed a significant linear trend among the ordered years from 2015 to 2020 (*p* = 0.011).

A total of 41 serotypes were identified among the 617 *Salmonella* isolates. Of which, *S.* Derby (107/617, 17.34%) was the most prevalent serotypes, followed by *S.* Enteritidis (81/617, 13.13%), *S.* Typhimurium (70/617, 11.35%), *S.* Rissen (33/617, 5.35%), *S.* Pullorum (32/617, 5.19%) and *S.* Indiana (31/617, 5.02%) (Figure 2D). In addition, 4 *Salmonella* isolates were identified as *S.* Typhimurium var. However, 19 (3.08%) *Salmonella* isolates were not serotyped.

### 3.2. Antibiotic susceptibility testing

**Figure 3:**
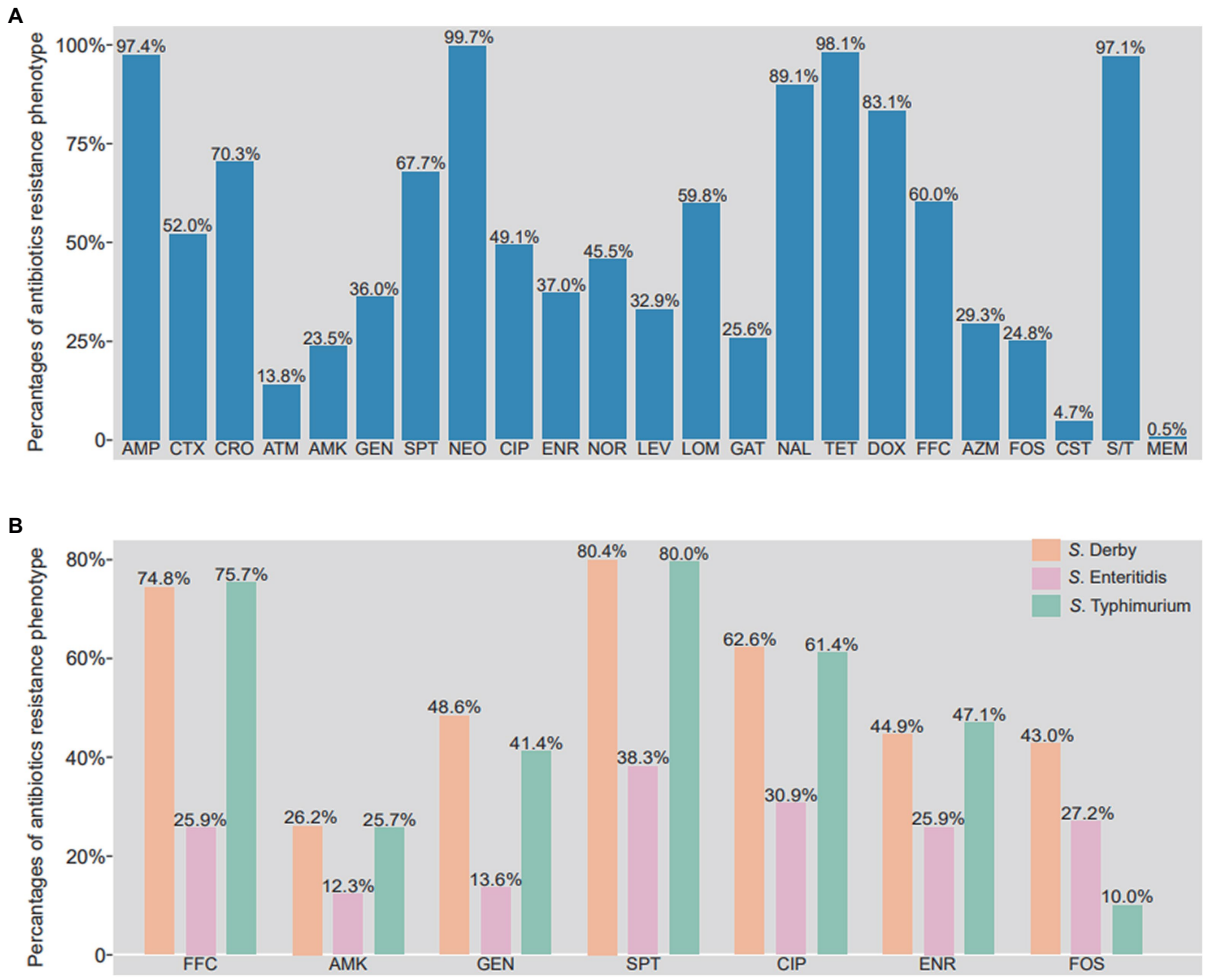
Phenotypic resistance profile of *Salmonella* isolates from food animals in China. **(A)** The percentage of antibiotics resistance phenotypes in 617 Salmonella isolates. **(B)** The different of antibiotics resistance phenotypes between *S*. Derby, *S*. Enteritidis and *S*. Typhimurium. AMP, ampicillin; CTX, cefotaxime; CRO, ceftriaxone; ATM, aztreonam; AMK, amikacin; GEN, gentamicin; SPT, spectinomycin; NEO, neomycin; CIP, ciprofloxacin; ENR, enrofloxacin; NOR, norfloxacin; LEV, levofloxacin; LOM, lomefloxacin; GAT, gatifloxacin; NAL, nalidixic acid; TET, tetracycline; DOX, doxycycline; FFC, florfenicol; AZM, azithromycin; FOS, phosphonomycin; CS, colistin; MEM, meropenem; S/T, trimethoprim/sulfamethoxazole.

As shown in Figure 3A, the majority of the *Salmonella* isolates were resistant to ampicillin (97.4%, 601/617), cefotaxime (52.0%, 321/617), ceftriaxone (70.3%, 434/617), spectinomycin (67.7%, 418/617), neomycin (99.7%, 617/617), lomefloxacin (59.8%, 369/617), nalidixic acid (89.1%, 550/617), tetracycline (98.1%, 605/617), doxycycline (83.1%, 513/617), florfenicol (60.0%, 370/617), and sulfadiazine/trimethoprim (97.1%, 599/617). In addition, a few of the *Salmonella* isolates were resistant to amikacin (23.5%, 145/617), gentamicin (36.0%, 222/617), ciprofloxacin (49.1%, 303/617), enrofloxacin (37.0%, 228/617), norfloxacin (45.5%, 281/617), levofloxacin (32.9%, 203/617), gatifloxacin (25.6%, 158/617), azithromycin (29.3%, 181/617), and fosfomycin (24.8%, 153/617). However, the low rates of resistance were observed in isolates to aztreonam (13.8%, 85/617), colistin (4.7%, 29/617), and meropenem (0.5%, 3/617).

It is worth noting that the resistant rates of amikacin, gentamicin, spectinomycin, florfenicol, ciprofloxacin, enrofloxacin in *S*. Derby and *S*. Typhimurium were higher than those in *S*. Enteritidis. Meanwhile, the resistant rate to fosfomycin from higher to lower was *S*. Derby (43.0%), *S*. Enteritidis (27.2%), and *S*. Typhimurium (10.0%) (Figure 3B). As shown in Supplementary Figure S1, 99.8% (616/617) *Salmonella* isolates were identified as multi-drug resistant, being resistant to more than 3 antibiotics. Some of isolates (9.7%, 60/617) were even resistant up to 12 antibiotics, two strains were resistant to 20 antibiotics, both of which were *S*. Typhimurium isolates. It is clear that *S*. Derby was mainly resistant to 9 (13/107, 12.1%), 11(14/107, 13.1%) and 14 antibiotics (14/107, 13.1%), *S*. Typhimurium was mainly resistant to 13 (10/74, 13.5%) and 15 (9/74, 12.2%) antibiotics, and *S*. Enteritidis was mainly resistant to 8 (10/81, 12.3%), 9 (13/81, 16.0%) and 11 (10/81, 12.3%) antibiotics (Supplementary Figure S2).

### 3.3. The detection of biofilm formation

In the biofilm formation test of 617 *Salmonella* isolates, 88.49% (546/617) of them were able to produce biofilm. Of which, 236 *Salmonella* isolates were characterized as strong biofilm producers (Supplementary Figure S3). The non-biofilm producers mainly belong to *S*. Derby (43.9%), yet the strong biofilm producers were majorly found as *S*. Enteritidis (48.2%) or *S*. Typhimurium (55.7%) (Supplementary Figure S4). Interestingly, the higher proportion of strong biofilm producers were observed in higher antibiotics resistant *Salmonella* isolates, such as: azithromycin (no biofilm 6.1%, strong biofilm 42.0%), colistin (no biofilm 0%, strong biofilm 69.0%), cefotaxime (no biofilm 9.7%, strong biofilm 37.4%), levofloxacin (no biofilm 6.9%, strong biofilm 45.3%), gatifloxacin (no biofilm 6.3%, strong biofilm 42.4%) (Supplementary Figure S5). The resistance rates of several antibiotics showed a significant correlation with the degree of biofilm production, *r* values were in azithromycin resistance (*r* = 0.997), colistin resistance (*r* = 0.933), cefotaxime resistance (*r* = 0.976), levofloxacin (*r* = 0.996), gatifloxacin resistance (*r* = 0.985).

### 3.4. Antimicrobial resistance genes genotyping

**Figure 4:**
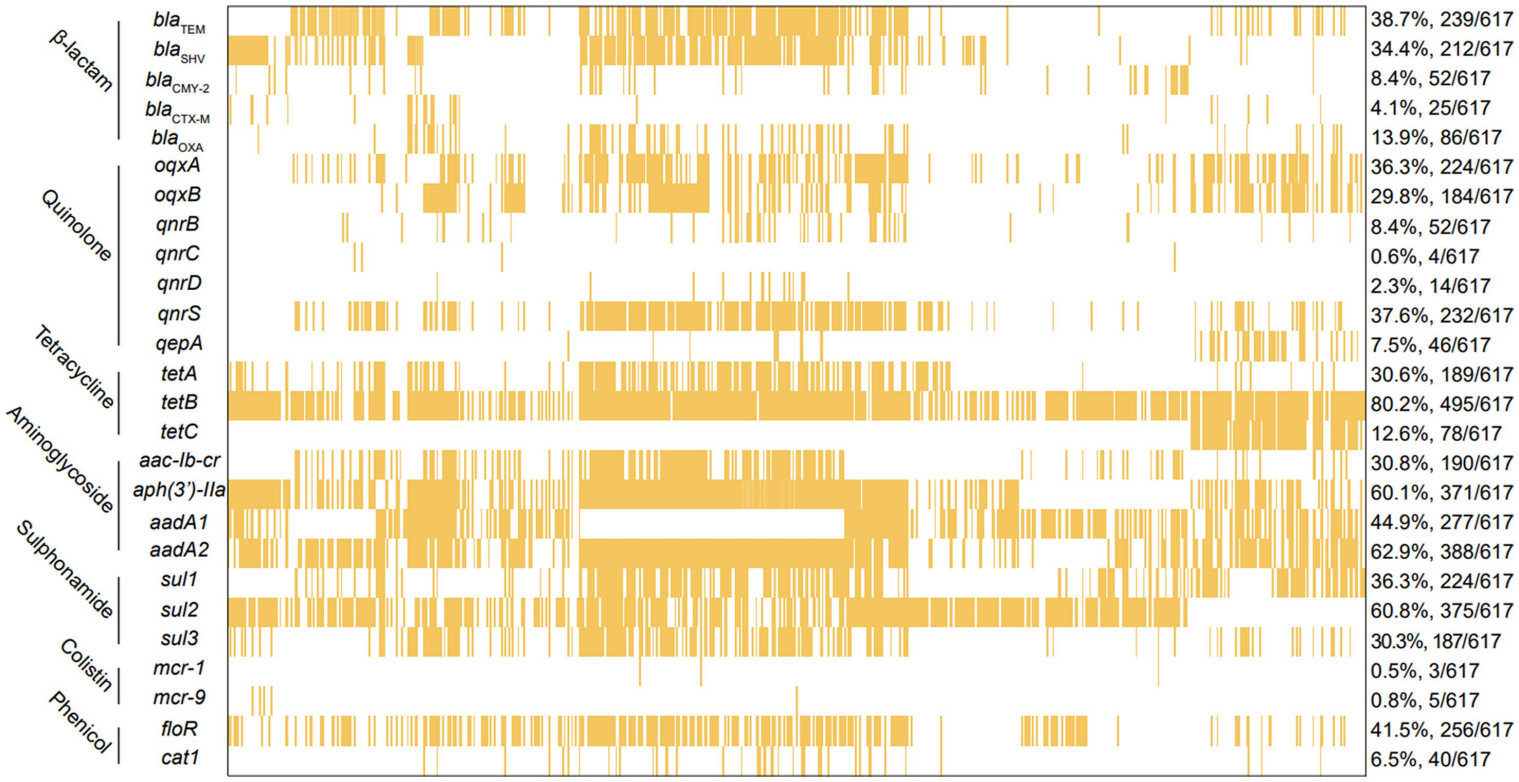
The analysis of ARGs among 617 *Salmonella* islolates from food animals in China. The yellow squares represent positivity for ARGs.

For the 617 *Salmonella* isolates in this study, a total 26 types of ARGs were identified, conferring resistance to seven classes of antibiotic including β-lactam, quinolone, tetracycline, aminoglycoside, sulfonamide, colistin, and phenicol (Figure 4). Among the 26 types of ARGs, 4 of them were highly prevalent, including tetracycline resistance gene *tetB* (80.2%, 495/617), sulfonamide resistance gene *sul2* (60.8%, 375/617), aminoglycoside resistance genes *aadA2* (62.9%, 388/617), and *aph(3′)-IIa* (60.1%, 371/617). In addition, 5 β-lactam resistance genes (*bla*_TEM_, *bla*_SHV_, *bla*_CMY-2_, *bla*_CTX-M_, and *bla*_OXA_) were detected and *bla*_TEM_ (38.7%, 239/617) was the most predominant genes responsible for β-lactam resistance. Here, 7 types of quinolone resistance genes were detected including *oqxA* (36.3%, 224/617), *oqxB* (29.8%, 184/617), *qnrB* (8.4%, 52/617), *qnrC* (0.6%, 4/617), *qnrD* (2.3%, 14/617), *qnrS* (37.6%, 232/617), and *qeqA* (7.5%, 46/617). Of note, some isolates were found to carry the coslitin resistance genes *mcr-1* (0.5%, 3/617) and *mcr-9* (0.8%, 5/617). However, the tigecycline resistance genes *tet(X3)*, *tet(X4)*, and *bla*_NDM_ were not detected among these *Salmonella* isolates.

Further analysis was performed on *Salmonella* isolates carrying more than three ARGs (Supplementary Figure S6). For these MDR *Salmonella* isolates, most of them carried 3 ~ 5 ARGs and the other could bear up to 6 ~ 14 ARGs. Notably, one strain carried 18 ARGs, four strains carried 17 ARGs and 12 strains carried 16 ARGs (Supplementary Figure S6). From the results of three major serotypes *Salmonella* strains carrying ARGs (Supplementary Figure S7), most of *S*. Derby carried 11 ~ 14 ARGs, most of *S*. Typhimurium carried 4 ~ 7 ARGs, most of *S*. Enteritidis carried 3 ~ 4 ARGs. Compare with *S*. Derby and *S*. Typhimurium, the *S*. Enteritidis harbored less ARGs.

### 3.5. Virulence genes profiling

**Figure 5:**
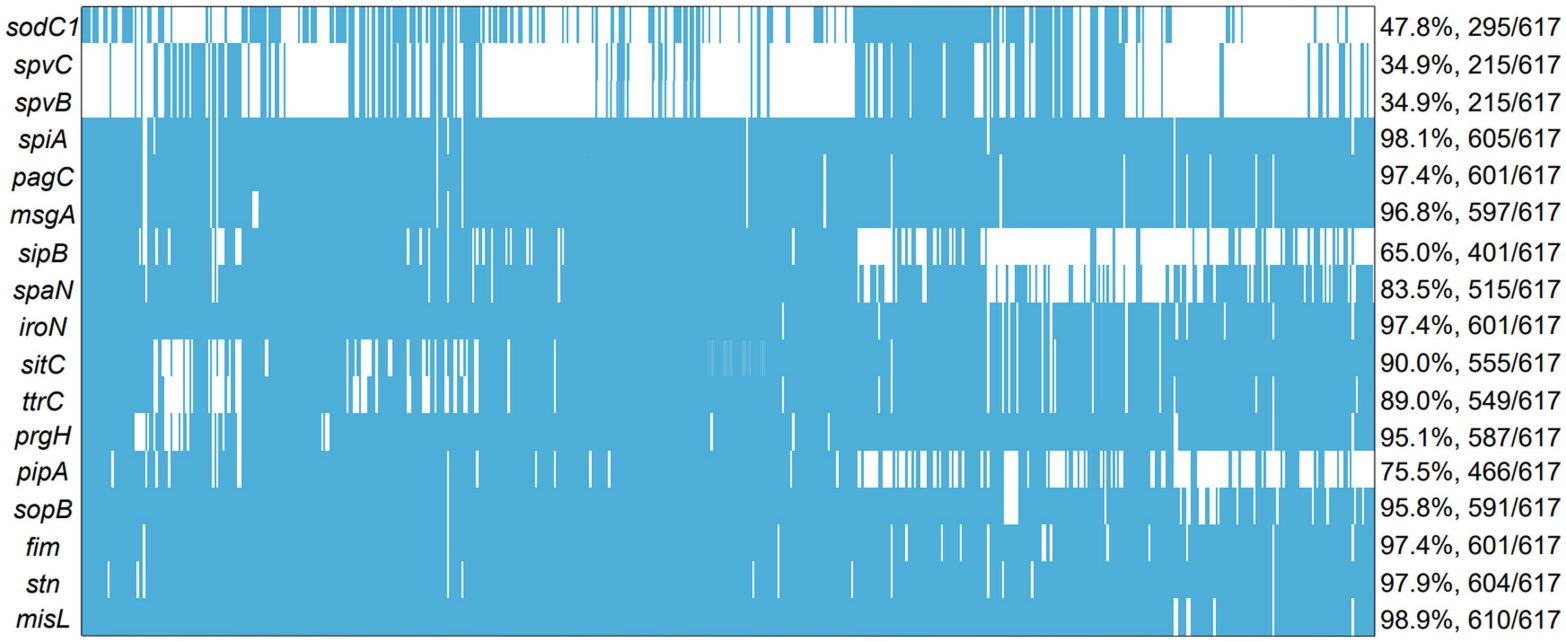
The analysis of virulence genes among 617 *Salmonella* islolates from food animals in China. The blue squares represent positivity for ARGs.

The frequencies of the 17 investigated virulence-associated genes were shown in Figure 5. It is worth noting that no isolate evaluated had all 17 genes. However, the prevalence of these genes was high since the lowest number of genes detected in one isolate was 5/17. Among the 17 virulence genes, 12 virulence genes were highly prevalent with presence of over 80% of total isolates, including *misL* (98.9%), *spiA* (98.1%), *stn* (97.9%), *pagC* (97.4%), *iroN* (97.4%), *fim* (97.4%), *msgA* (96.8%), *sopB* (95.8%), *prgH* (95.1%), *sitC* (90.0%), *ttrC* (89.0%), *span* (83.5%). The other 5 virulence genes, such as *pipA* (75.5%), *sipB* (65.0%)*, sodC1* (47.8%)*, spvC* (34.9%)*, spvB* (34.9%), with minor presence are detailed in Figure 5. The highest prevalence of gene detected was *misL* (98.9%), but he lowest prevalence of gene detected was *spvC and spvB* (34.9%). Notably, the number of virulence genes in *S*. Derby and *S*. Enteritidis were significantly higher than those in *S*. Typhimurium (*p* < 0.005). However, the numbers of virulence genes were similar in *S*. Derby and *S*. Enteritidis (Supplementary Figure S8).

## 4. Discussion

In this study, a total of 617 *Salmonella* isolates were obtained from 4 major food animals in 23 provinces, China. As shown in the section above, animal products in Guangdong and Shandong demonstrated more *Salmonella* contamination In a previous study, we found that food animals, especially the chickens, pigs, ducks, and geese, in Shandong and Guangdong have been massively contaminated by antibiotics-resistant Enterobacteriaceae (Wang et al., 2021). Therefore, we initiated this study to further investigate the *Salmonella* colonization on these food animals. Similar to previous reports, *Salmonella* isolates have been frequently recovered from foods like chicken, eggs, pork, duck, beef, peanut, and vegetables (Viana et al., 2019; von Hertwig et al., 2019; Chen et al., 2020; Hai et al., 2020; Yang et al., 2020b). As indicated in this study, prevalence of *Salmonella* has accumulated significantly since 2015 by 5.51% to 2020 by 27.23%. This interestingly increasing trend in *Salmonella* prevalence is similar to human non-typhoidal *Salmonella* (NTS) infections from 2004 to 2016 by 17% (Bharat et al., 2021).

There were 41 serotypes identified among the 617 *Salmonella* isolates, from which *S.* Derby, was the most common serovar followed by *S.* Enteritidis and *S.* Typhimurium. *S.* Derby were also previously found to be the predominant serovar in retail meat, particularly in pork meat (Xu et al., 2020). In several provinces of China, this serovar was also considered as the most prevalent *Salmonella* species from pig borne food (Jiang et al., 2021). Nonetheless, *S.* Enteritidis, and *S.* Typhimurium were the most common non-typhoidal *Salmonella* serovars in surveillance for invasive *Salmonella* disease from 2002 to 2018 (Still et al., 2020). In another study, 35 serovars were identified among 667 *Salmonella* isolates from retail poultry meat, and predominant serovars were *S.* Enteritidis, *S.* Indiana and *S.* Typhimurium (Yang et al., 2020a). Also, there were 19 isolates whose serotypes were not clearly determined, indicating that there were possibly new serotypes in development and the causes of the contamination is complicated (Xu et al., 2020).

Widespread and improper usage of antimicrobials accelerate the development and spread of AMR (Raufu et al., 2021). Although the presence of infection caused by antimicrobial resistant *Salmonella* reported worldwide, the cases in developing countries is remarkably increasing at an alarming rate (Jiang et al., 2021). Antibiotics selected for this study were all first-line drugs currently in clinical use in veterinary medicine. Our results suggested that antimicrobial resistance was present in the majority of *Salmonella* isolates, 80% isolates were resistant to ampicillin, neomycin, nalidixic acid, tetracycline, doxycycline, and sulfadiazine/trimethoprim. The outcome was consistent with those reports (Fang et al., 2019; Asefa Kebede and Duga, 2022; Borah et al., 2022; Hassena et al., 2022). Noteworthy, meropenem resistance appeared in fewer isolates (0.5%) in our study. These outcome was in disagreement with other reports that indicated no resistance for colistin, nalidixic acid, meropenem, gentamicin, florfenicol and chloramphenicol in Australian (Abraham et al., 2022), no resistance for meropenem in Eastern China (Tang et al., 2022), fewer resistance for ampicillin and sulfonamide-trimethoprim in Ethiopia (Asefa Kebede and Duga, 2022), These disagreement may result from geographical and biological differences exist among various strains. In addition, colistin is considered one of the last resort for the treatment of multidrug-resistant *Enterobacteriaceae* (Elbediwi et al., 2019), 4.7% of *Salmonella* isolates showed resistance to colistin in our study, which was consistent with other reports (Li et al., 2022; Tang et al., 2022) from poultry and pigs. In present study, more antimicrobial resistance was identified in *S*. Derby and *S*. Typhimurium than those in *S*. Enteritidis. It is different from a previous study which characterized the *S*. Indiana with most enriched antimicrobial resistance genes among all serovars (Yang et al., 2020a). In this study, 99.8% of the *Salmonella* isolates were MDR, which was consistent with Tang’s report (Tang et al., 2022). The majority of *Salmonella* isolates were resistant to 8 ~ 12 antibiotics, highlighting the worsening situation of multidrug resistance development in *Salmonella* in China. Therefore, the continuous surveillance of antimicrobial resistance in *Salmonella* and political implements are conducive to safeguard consumer health (Chen et al., 2020).

Biofilm formation is a crucial strategy for *Salmonella* survival under unfavorable environmental conditions (Merino et al., 2019). Studies have found a range of bacterial cell surface components such as cellulose, flagella and fimbriae contributing to the attachment of *Salmonella* to different surfaces (Kroupitski et al., 2009). In this study, we found that 88.49% of *Salmonella* isolates were able to produce biofilms at different degree. A total 236 *Salmonella* isolates (38.3%) were strong biofilm producer. Some studies show that all of the *Salmonella* isolates from the chain of beef production and retail were able to form biofilm (Yin et al., 2018; Manafi et al., 2020). Also, we found that the detection rates of strong biofilm producer were higher in *S*. Enteritidis (48.2%) and *S*. Typhimurium (55.7%) isolates. This finding is consistent with a previous study that *S*. Enteritidis was the strongest biofilm producer (Manafi et al., 2020). The enhanced biofilm producing ability demonstrated high co-occurrence with the resistance to azithromycin, colistin, cefotaxime levofloxacin, gatifloxacin. These may be due to co-localization of genes encoding biofilm and antibiotics resistance in *Salmonella* isolates (Shi et al., 2018). Voss-Rech et al. reported that 65% of the isolates showed the ability to produce biofilm in Brazil (Voss-Rech et al., 2022), and Manafi et al. reported that all isolates were able to form biofilm (75.86% moderate and 24.14% strong) (Manafi et al., 2020). These previous results are consistent with our findings, showed that antimicrobial resistance may correlate with biofilm formation. Biofilm formation will enhance resistance to antibiotics and disinfectants, enhance the ability of *Salmonella* to survive in animals and the environment, leading to big harm to animals (Dai et al., 2021). This study underlined the ability of *Salmonella* to contaminate food possibly attributed by their biofilm producing capability. Therefore, it is important to study the mechanism of biofilm formation to prevent the spread and infection of *Salmonella*.

*Salmonella* harbor diverse antibiotic resistance genes along with mobile genetic elements, which accelerate the dissemination of resistance to other serotypes or even bacteria of different genera (Sharma et al., 2019). Five β-lactam resistance genes were detected among these *Salmonella* isolates, and *bla*_TEM_ was the most predominant, which is consistent with prior studies that *bla*_TEM_ was the most frequent genotype to confer the β-lactam resistance to *Salmonella* from retail chicken meat, poultry, pig and humans (Djeffal et al., 2017; Trongjit et al., 2017; Sharma et al., 2019). Seven types of quinolone resistance genes were detected including *oqxAB*, *qnrBCDS*, and *qeqA*. The co-existence of quinolone resistance genes with other clinically important ARGs including ESBL genes was observed in current and previous study (Fang et al., 2019). In this study, *tetB* and *sul2* were the most frequent in tetracycline and sulfonamide resistance genes, respectively. Contrary to previous publications, Sharma and coworkers reported that *tetA* and *sul1* were the most frequent resistance genes to protect *Salmonella* from bactericidal effect of tetracycline and sulfonamide antibiotics (Sharma et al., 2019). The studies on the number of ARGs carried by strains, results showed that all strains carried more than three ARGs, and one strain carried 18 ARGs, which warranted in-depth study. These results are consistent with the reported results (Akinyemi et al., 2011; Ammar et al., 2016a; Abraham et al., 2022; Hassena et al., 2022; Kong-Ngoen et al., 2022; Tang et al., 2022), which all concluded that *Salmonella* carries multiple ARGs. The results of our analysis for the three main prevalent serotypes carried ARGs, showed that *S*. Derby mainly carried 11 ~ 14 genes, *S*. Typhimurium mainly carried 4 ~ 7 genes, and *S*. Enteritidis mainly carried 3 ~ 4 ARGs. This result revealed that there is no correlation between serotype and ARGs. The occurrence of this may be related to the resistance genes mainly associated with antibiotic resistance (Ammar et al., 2016a; Abraham et al., 2022).

The currently known virulence factors of *Salmonella* include toxin production, capsule, flagellum, fimbriae, secretory system and other factors which are involved in various stages of infection (Sedrakyan et al., 2022). *Salmonella* virulence factors encode products to assist invading procedures in the host (Raufu et al., 2021). *PipA* was an effector protein, which redundantly target components of the NF signaling pathway to cause inflammation (Sun et al., 2016). *Salmonella* invasion protein B (*Sip*B) initiates the invasion process which belongs to *Salmonella* type 3 secretion system (Chen et al., 2018). *sodC1* and *sodC2* were two genes encoding periplasmic superoxide dismutase, locating on lambdoid prophage and chromosome, respectively. These genes contribute to *Salmonella* virulence by protecting bacteria from superoxide radicals generated by host’s phagocytes (Ammendola et al., 2005). *SpvB* presented on the plasmid and facilitated *Salmonella* survival and replication within macrophages *via* perturbing cellular iron metabolism (Deng et al., 2021). The role of some of these virulence-associated plasmids in the dissemination of increased virulence in food-animal environments and humans (Ammar et al., 2016b; Khajanchi and Foley, 2022). While the main differences between isolates were attributed to the serotype-specific diversity of virulence genes, SPIs, virulence plasmids, and phages (Hassena et al., 2022). The presence of some virulence genes was serotype specific (Sedrakyan et al., 2022). In this study, 17 virulence genes were identified among these *Salmonella* isolates. The results suggested that the isolates regularly harboring most of virulence genes selected in this study with a detection rate of over 80%. This is in agreement with the findings in previous studies (Hai et al., 2020; Borah et al., 2022; Siddiky et al., 2022). By contrast, 6 virulence genes (*pipA*, *sipB*, *sodC*, *spvB* and *spvC*) were detected at lower presence. The diversity of virulence genes may provide important characterization clues for the further study of *Salmonella* pathogenicity (Sedrakyan et al., 2022). In this study, the high detection rate of these virulence genes may explain why *Salmonella* infection is prone to cause significant morbidity or mortality in animals. The action mechanism of virulence genes in *Salmonella* needs to be further investigated. Continuous surveillance of the prevalence, resistance and virulence of *Salmonella* in food animals will greatly enhance the control surveillance and future outbreak investigation of the infection.

## 5. Conclusion

In conclusion, this study investigated the prevalence and distribution of *Salmonella* isolates from food animals in China. A total of 41 serotypes were identified, and *S.* Derby, *S.* Enteritidis, and *S.* Typhimurium were the most prevalent serotypes. The high rates of antimicrobial resistance were observed among the majority of the *Salmonella* isolates, and biofilm formation ability can enhance the resistance of *Salmonella* to antibiotics. In addition, these isolates carried abundant antibiotics resistance genes and virulence genes. This study provided useful information regarding the epidemiological characteristics of *Salmonella* in the food animals in China and may help the policy-making to better control the fast development *Salmonella* contaminations China.

## Data availability statement

The original contributions presented in the study are included in the article/Supplementary material, further inquiries can be directed to the corresponding author/s.

## Author contributions

YZ and XL designed and coordinated this research and drafted the manuscript. LG conducted experiments. YL, JS, and HR modified the manuscript. TX, LW and WL carried out the data analysis. KL and WJ conceived of this study. YL and XD revised the manuscript. All authors contributed to the article and approved the submitted version.

## Funding

This work was jointly supported by grants from PhD Fund of Qingdao Agricultural University (663-1119017), Shandong Provincial the Development of new raw materials and preparations for Animal Respiratory Disease Control under Grant (662-2321018), Taishan Industrial Experts Program (TS20220701), Qingdao Science and Technology for the People Demonstration Special-Creation of monitoring and early warning platform and demonstration of purification and prevention and control for important animal diseases (2023), Innovative Research Groups of the National Natural Science Foundation of China(32121004), Local Innovative and Research Teams Project of Guangdong Pearl River Talents Program (2019BT02N054), Program for Changjiang Scholars and Innovative Research Team in University of Ministry of Education of China (IRT_17R39) and Innovation Team Project of Guang dong University (2019KCXTD001).

## Conflict of interest

The authors declare that the research was conducted in the absence of any commercial or financial relationships that could be construed as a potential conflict of interest.

## Publisher’s note

All claims expressed in this article are solely those of the authors and do not necessarily represent those of their affiliated organizations, or those of the publisher, the editors and the reviewers. Any product that may be evaluated in this article, or claim that may be made by its manufacturer, is not guaranteed or endorsed by the publisher.
