# Supplementary material for: Comprehensive profiling of serotypes, antimicrobial resistance and virulence of Salmonella isolates from food animals in China, 2015–2021
Source: Front Microbiol. 2023 Apr 4;14:1133241. doi: 10.3389/fmicb.2023.1133241 (PMC10110913; doi:10.3389/fmicb.2023.1133241)
Supplement: Supplementary file 1 [file Table_1.docx]

**Supplementary Tables:**

**Table S1. The primers of 32 antibiotic resistance genes.**

| **Genes** | | | **Primers** | **Length(bp)** | **References** |
| --- | --- | --- | --- | --- | --- |
| Fluoroquinolones | *qnrA* | qnrA-F | TCAGCAAGAGGATTTCTCA | 627 | (Chen, et al., 2020) |
|  |  | qnrA-R | GGCAGCACTATTACTCCCA |  |  |
|  | *qnrB* | qnrB-F | GATCGTGAAAGCCAGAAAGG | 469 | (Chen, et al., 2020) |
|  |  | qnrB-R | ACGATGCCTGGTAGTTGTCC |  |  |
|  | *qnrC* | qnrC-F | GGGTTGTACATTTATTGAATC | 447 | (Chen, et al., 2020) |
|  |  | qnrC-R | TCCACTTTACGAGGTTCT |  |  |
|  | *qnrD* | qnrD-F | TCCACTTTACGAGGTTCT | 556 | (Chen, et al., 2020) |
|  |  | qnrD-R | CGAGATCAATTTACGGG |  |  |
|  | *qnrS* | qnrS-F | ACGACATTCGTCAACTGCAA | 417 | (Chen, et al., 2020) |
|  |  | qnrS-R | TAAATTGGCACCCTGTAGGC |  |  |
|  | *aac(6')-Ib-cr* | Aac(6')-Ib-cr-F | TTGCGATGCTCTATGAGTGGCTA | 482 | (Chen, et al., 2020) |
|  |  | Aac(6')-Ib-cr-R | CTCGAATGCCTGGCGTGTTT |  |  |
|  | *qepA* | qepA-F | GCAGGTCCAGCAGCGGGTAG | 199 | (Fang, et al., 2019) |
|  |  | qepA-R | CTTCCTGCC CGAGTATCGTG |  |  |
|  | *oqxA* | oqxA-F | GACAGCGTCGCACAGAATG | 339 | (Fang, et al., 2019) |
|  |  | oqxA-R | GGAGACGAGGTTGGTATGGA |  |  |
|  | *oqxB* | oqxB-F | CGAAGAAAGACCTCCCTACCC | 240 | (Fang, et al., 2019) |
|  |  | oqxB-R | CGCCGCCAATGAGATACA |  |  |
| β-lactams | *CTX* | CTX-M-F | GAGTTTCCCCATTCCGTTTC | 909 | (Chen, et al., 2020) |
|  |  | CTX-M-R | CAGAATAAGGAATCCCATGGTT |  |  |
|  | *TEM* | TEM-F | ATGAGTATTCAACATTTCCG | 964 | (Chen, et al., 2020) |
|  |  | TEM-R | ACCAATGCTTAATCAGTGAG |  |  |
|  | *SHV* | SHV-F | TTCGCCTGTGTATTATCTCCCTG | 854 | (Chen, et al., 2020) |
|  |  | SHV-R | TTAGCGTTGCCAGTGCTCG |  |  |
|  | *ACC* | ACC-F | AGCCTCAGCAGCCGGTTAC | 818 | (Maravić, et al., 2013) |
|  |  | ACC-R | GAAGCCGTTAGTTGATCCGG |  |  |
|  | *OXA* | OXA-F | ACCAGATTCAACTTTCAA | 590 | (Maravić, et al., 2013) |
|  |  | OXA-R | TCTTGGCTTTTATGCTTG |  |  |
|  | *VEB* | VEB-F | GATAGGAGTACAGACATATG | 914 | (Maravić, et al., 2013) |
|  |  | VEB-R | TTTATTCAAATAGTAATTCCACG |  |  |
|  | *CMY*-2 | blaCMY-2-F | TGGCCGTTGCCGTTATCTAC | 868 | (Maravić, et al., 2013) |
|  |  | blaCMY-2-R | CCCGTTTTATGCACCCATGA |  |  |
| Chloramphenicol | *floR* | floR-F | ATCCAACTCACGTTGAGCC | 868 | (Chen, et al., 2020) |
|  |  | floR-R | TTGGATGCAGAAGTAGAACG |  |  |
|  | *cat1* | cat1-F | AGTGGAATAACGAACGAGC | 470 | (Chen, et al., 2020) |
|  |  | cat1-R | TCAGCAAGCGATATACGCAG |  |  |
| Sulfonamides | *sul1* | sul1-F | CTTCGATGAGAGCCGGCGGC | 437 | (Chen, et al., 2020) |
|  |  | sul1-R | GCAAGGCGGAAACCCGCGCC |  |  |
|  | *sul2* | sul2-F | GCGCTCAAGGCAGATGGCATT | 285 | (Chen, et al., 2020) |
|  |  | sul2-R | GCGTTTGATACCGGCACCCGT |  |  |
|  | *sul3* | sul3-F | AGATGTGATTGATTTGGGAGC | 443 | (Chen, et al., 2020) |
|  |  | sul3-R | TAGTTGTTTCTGGATTAGAGCCT |  |  |
| Tetracycline | *tetA* | tetA-F | GTAATTCTGAGCACTGTCGC | 956 | (Chen, et al., 2020) |
|  |  | tetA-R | CTGCCTGGACAACATTGCTT |  |  |
|  | *tetB* | tetB-F | GAGACGCAATCGAATTCGG | 228 | (Chen, et al., 2020) |
|  |  | tetB-R | TTTAGTGGCTATTCTTCCTGCC |  |  |
|  | *tetC* | tetC-F | CTTGAGAGCCTTCAACCCAG | 418 | (Chen, et al., 2020) |
|  |  | tetC-R | ATGGTCGTCATCTACCTGCC |  |  |
|  | *tetX* | tetX-F | GAAAGAGACAACGACCGAGAG | 199 | (Sun, et al., 2019) |
|  |  | tetX-R | ACACCCATTGGTAAGGCTAAG |  |  |
|  | *tetX3* | tetX3-F | TAATGGCGGGACATCAGG | 205 | (Sun, et al., 2019) |
|  |  | tetX3-R | AGGCGACATCAAATGAGCAG |  |  |
|  | *tetX4* | tetX4-F | TGTCCTGCAAAAGGAGGCAT | 431 | (Sun, et al., 2019) |
|  |  | tetX4-R | ACCGACACGGAAGTTGAAGA |  |  |
| Aminoglycosides | *aadA1* | aadA1-F | TTTGCTGGTTACGGTGAC | 497 | (Chen, et al., 2020) |
|  |  | aadA1-R | GCTCCATTGCCCAGTCG |  |  |
|  | *aadA2* | aadA2-F | GGTGCTAAGCGTCATTGAGC | 470 | (Chen, et al., 2020) |
|  |  | aadA2-R | GCTTCAAGGTTTCCCTCAGC |  |  |
|  | *aph(3')-Ⅱa* | aph(3')-Ⅱa-F | TCTGAAACATGGCAAAGGTAG | 582 | (Chen, et al., 2020) |
|  |  | aph(3')-Ⅱa-R | AGCCGTTTCTGTAATGAAGGA |  |  |
| Macrolides | *NDM* | NDM-F | GCACACTTCCTATCTCGACATG | 465 | (Han, Liu, Cui, Cheng, & Jiang, 2020) |
|  |  | NDM-R | GCCTTGCTGTCCTTGATCAG |  |  |
|  | *mcr* | mcr-F | CGGTCAGTCCGTTTGTTC | 309 | (Han, et al., 2020) |
|  |  | mcr-R | CTTGGTCGGTCTGTAGGG |  |  |

**Table S2. The primers of SPI and virulence genes.**

| **Genes** | | **Primers** | **Length(bp)** | **References** |
| --- | --- | --- | --- | --- |
| *spvC* | spvC-F | TATGATGGGGCGGAAA | 252 | (Parvathi, Vijayan, Murali, & Chandran, 2011) |
|  | spvC-R | AGGCTAACACGGGCTT |  |  |
| *spvB* | spvB-F | CCTGATGTTCCACCACTTTC | 590 | (Parvathi, et al., 2011) |
|  | spvB-R | ATGCCTTATCTGGCGATGT |  |  |
| *sodC1* | sodC1-F | CAGTTGTTCAGCAATGGCAGAG | 403 | (Sánchez-Jiménez, Cardona-Castro, Canu, Uzzau, & Rubino, 2010) |
|  | sodC1-R | CATCAATGAGTGACCTTTCAGTTC |  |  |
| *spiA* | spiA-F | CCAGGGGTCGTTAGTGTATTGCGTGAGATG | 550 | (Skyberg, Logue, & Nolan, 2006) |
|  | spiA-R | CGCGTAACAAAGAACCCGTAGTGATGGATT |  |  |
| *pagC* | pagC-F | CGCCTTTTCCGTGGGGTATGC | 454 | (Skyberg, et al., 2006) |
|  | pagC-R | GAAGCCGTTTATTTTTGTAGAGGAGATGTT |  |  |
| *msgA* | msgA-F | GCCAGGCGCACGCGAAATCATCC | 189 | (Skyberg, et al., 2006) |
|  | msgA-R | GCGACCAGCCACATATCAGCCTCTTCAAAC |  |  |
| *sipB* | sipB-F | GGACGCCGCCCGGGAAAAACTCTC | 875 | (Skyberg, et al., 2006) |
|  | sipB-R | ACACTCCCGTCGCCGCCTTCACAA |  |  |
| *spaN* | spaN-F | AAAAGCCGTGGAATCCGTTAGTGAAGT | 504 | (Skyberg, et al., 2006) |
|  | spaN-R | CAGCGCTGGGGATTACCGTTTTG |  |  |
| *iroN* | iroN-F | ACTGGCACGGCTCGCTGTCGCTCTAT | 1205 | (Skyberg, et al., 2006) |
|  | iroN-R | CGCTTTACCGCCGTTCTGCCACTGC |  |  |
| *sitC* | sitC-F | CAGTATATGCTCAACGCGATGTGGGTCTCC | 768 | (Skyberg, et al., 2006) |
|  | sitC-R | CGGGGCGAAAATAAAGGCTGTGATGAAC |  |  |
| *ttrC* | ttrC-F | GTGGGCGGTACAATATTTCTTTT | 920 | (Sánchez-Jiménez, et al., 2010) |
|  | ttrC-R | TCACGAATAATAATCAGTAGCGC |  |  |
| *prgH* | prgH-F | GCCCGAGCAGCCTGAGAAGTTAGAAA | 756 | (Skyberg, et al., 2006) |
|  | *prgH-R* | TGAAATGAGCGCCCCTTGAGCCAGTC |  |  |
| *pipA* | pipA-F | CTCTTGGATGATTTTCTTCTTTA | 406 | (Parvathi, et al., 2011) |
|  | pipA-R | CTTATCTCAGGCGCGGGTGG |  |  |
| *sopB* | sopB-F | CGGACCGGCCAGCAACAAAACAAGAAGAAG | 220 | (Skyberg, et al., 2006) |
|  | sopB-R | TAGTGATGCCCGTTATGCGTGAGTGTATT |  |  |
| *misL* | misL-F | GTCGGCGAATGCCGCGAATA | 561 | (Sánchez-Jiménez, et al., 2010) |
|  | misL-R | GCGCTGTTAACGCTAATAGT |  |  |
| *fim* | fim-F | CTATTGCGAGTCTGAT | 344 | (Akinyemi, Iwalokun, Foli, Oshodi, & Coker, 2011) |
|  | fim-R | GTAGTGCTATTGTCCG |  |  |
| *stn* | stn-F | CAACCAGATAGTAAAGACCG | 234 | (Akinyemi, et al., 2011) |
|  | stn-R | ATTAGCGTAGAGGCAAAAGA |  |  |

Akinyemi, K. O., Iwalokun, B. A., Foli, F., Oshodi, K., & Coker, A. O. (2011). Prevalence of multiple drug resistance and screening of enterotoxin (stn) gene in Salmonella enterica serovars from water sources in Lagos, Nigeria. *Public Health, 125*(2), 65-71.

Chen, Z., Bai, J., Wang, S., Zhang, X., Zhan, Z., Shen, H., Zhang, H., Wen, J., Gao, Y., Liao, M., & Zhang, J. (2020). Prevalence, Antimicrobial Resistance, Virulence Genes and Genetic Diversity of Salmonella Isolated from Retail Duck Meat in Southern China. *Microorganisms, 8*(3).

Fang, L. X., Deng, G. H., Jiang, Q., Cen, D. J., Yang, R. S., Feng, Y. Y., Xia, J., Sun, J., Liu, Y. H., Zhang, Q., & Liao, X. P. (2019). Clonal expansion and horizontal transmission of epidemic F2:A1:B1 plasmids involved in co-spread of rmtB with qepA and blaCTX-M-27 in extensively drug-resistant Salmonella enterica serovar Indiana isolates. *J Antimicrob Chemother, 74*(2), 334-341.

Han, H., Liu, W., Cui, X., Cheng, X., & Jiang, X. (2020). Co-Existence of mcr-1 and bla (NDM-5) in an Escherichia coli Strain Isolated from the Pharmaceutical Industry, WWTP. *Infect Drug Resist, 13*, 851-854.

Maravić, A., Skočibušić, M., Samanić, I., Fredotović, Z., Cvjetan, S., Jutronić, M., & Puizina, J. (2013). Aeromonas spp. simultaneously harbouring bla(CTX-M-15), bla(SHV-12), bla(PER-1) and bla(FOX-2), in wild-growing Mediterranean mussel (Mytilus galloprovincialis) from Adriatic Sea, Croatia. *Int J Food Microbiol, 166*(2), 301-308.

Parvathi, A., Vijayan, J., Murali, G., & Chandran, P. (2011). Comparative virulence genotyping and antimicrobial susceptibility profiling of environmental and clinical Salmonella enterica from Cochin, India. *Curr Microbiol, 62*(1), 21-26.

Sánchez-Jiménez, M. M., Cardona-Castro, N., Canu, N., Uzzau, S., & Rubino, S. (2010). Distribution of pathogenicity islands among Colombian isolates of Salmonella. *J Infect Dev Ctries, 4*(9), 555-559.

Skyberg, J. A., Logue, C. M., & Nolan, L. K. (2006). Virulence genotyping of Salmonella spp. with multiplex PCR. *Avian Dis, 50*(1), 77-81.

Sun, J., Chen, C., Cui, C. Y., Zhang, Y., Liu, X., Cui, Z. H., Ma, X. Y., Feng, Y., Fang, L. X., Lian, X. L., Zhang, R. M., Tang, Y. Z., Zhang, K. X., Liu, H. M., Zhuang, Z. H., Zhou, S. D., Lv, J. N., Du, H., Huang, B., Yu, F. Y., Mathema, B., Kreiswirth, B. N., Liao, X. P., Chen, L., & Liu, Y. H. (2019). Plasmid-encoded tet(X) genes that confer high-level tigecycline resistance in Escherichia coli. *Nat Microbiol, 4*(9), 1457-1464.
