# Supplementary material for: Comprehensive profiling of serotypes, antimicrobial resistance and virulence of Salmonella isolates from food animals in China, 2015–2021
Source: Front Microbiol. 2023 Apr 4;14:1133241. doi: 10.3389/fmicb.2023.1133241 (PMC10110913; doi:10.3389/fmicb.2023.1133241)
Supplement: Supplementary file 2 [file Data_Sheet_1.docx]

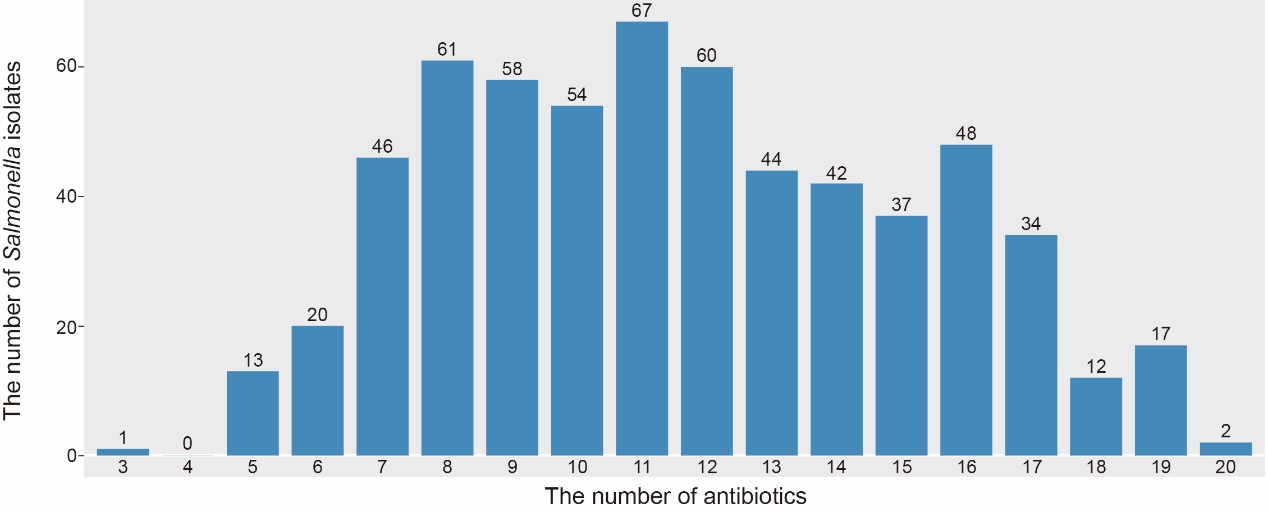


**Figure S1.** The number of MDR *Salmonella* isolates from food animals in China.

**Figure S2.** The number of MDR *Salmonella* isolates in *S*. Derby, *S*. Enteridis, and *S*. Typimurium isolates, respectively.

**
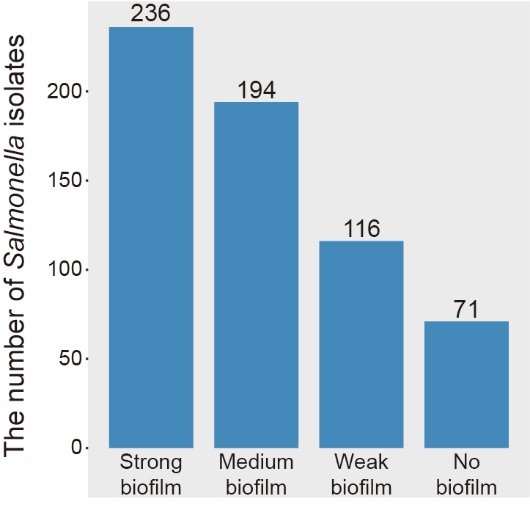
**

**Figure S3.** The analysis of biofilm formation ability of 617 Salmonella isolates from food animals in China.


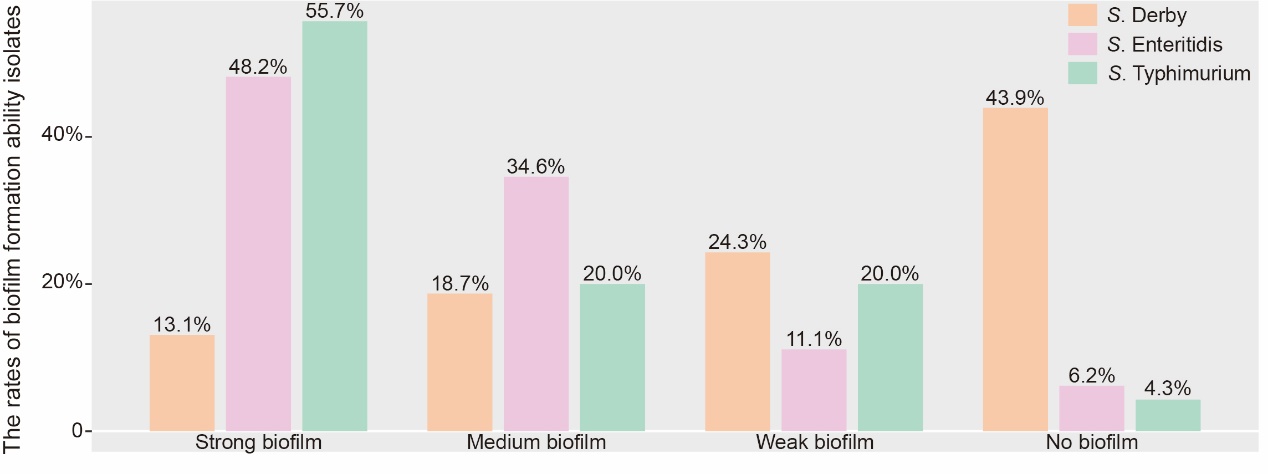


**Figure S4.** The analysis of biofilm formation ability of 617 *Salmonella* isolates from food animals in China.


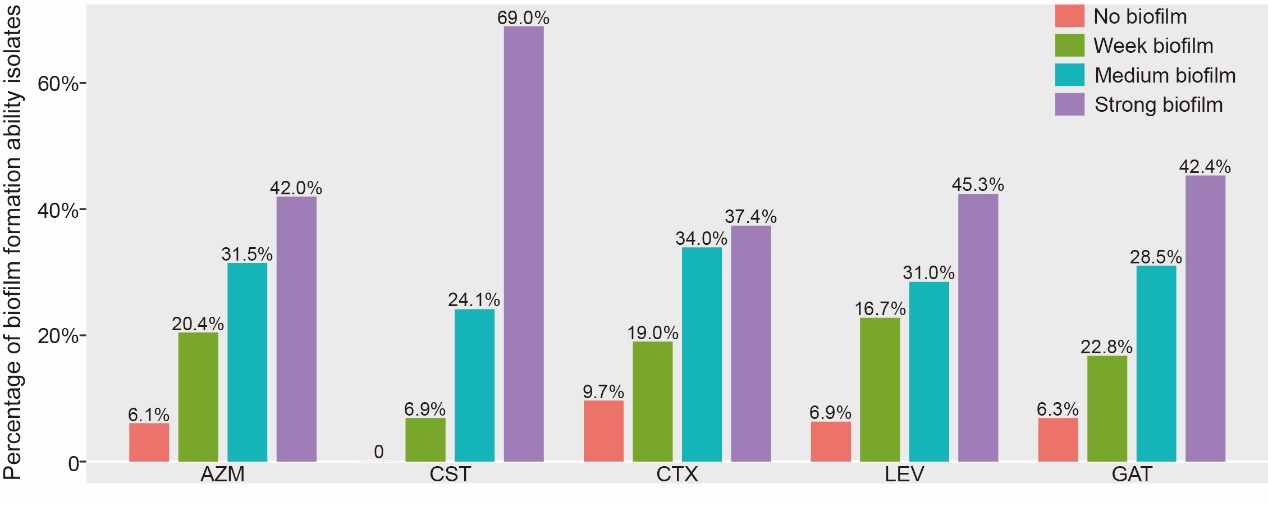


**Figure S5.** Comparison analysis of biofilm formation ability of antibiotics resistant *Salmonella* isolates from food animals in China. AZM, azithromycin; CST, colistin; CTX, cefotaxime; LEV, levofloxacin; GAT, gatifloxacin.


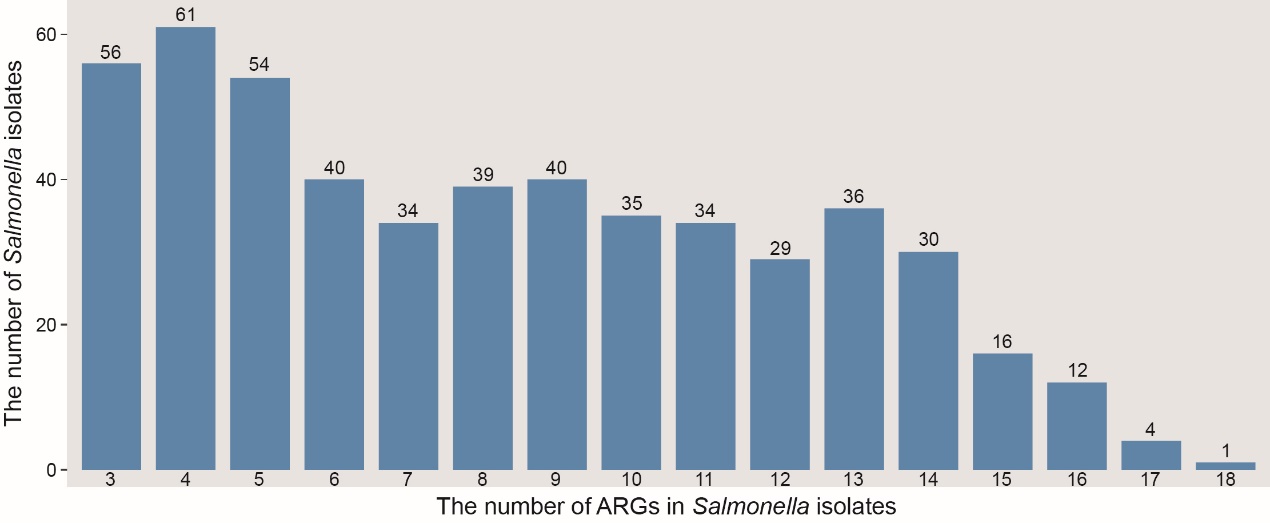


**Figure S6.** The number of isolates carrying different number of ARGs in Salmonella isolates collected from food animals across different provinces of China.


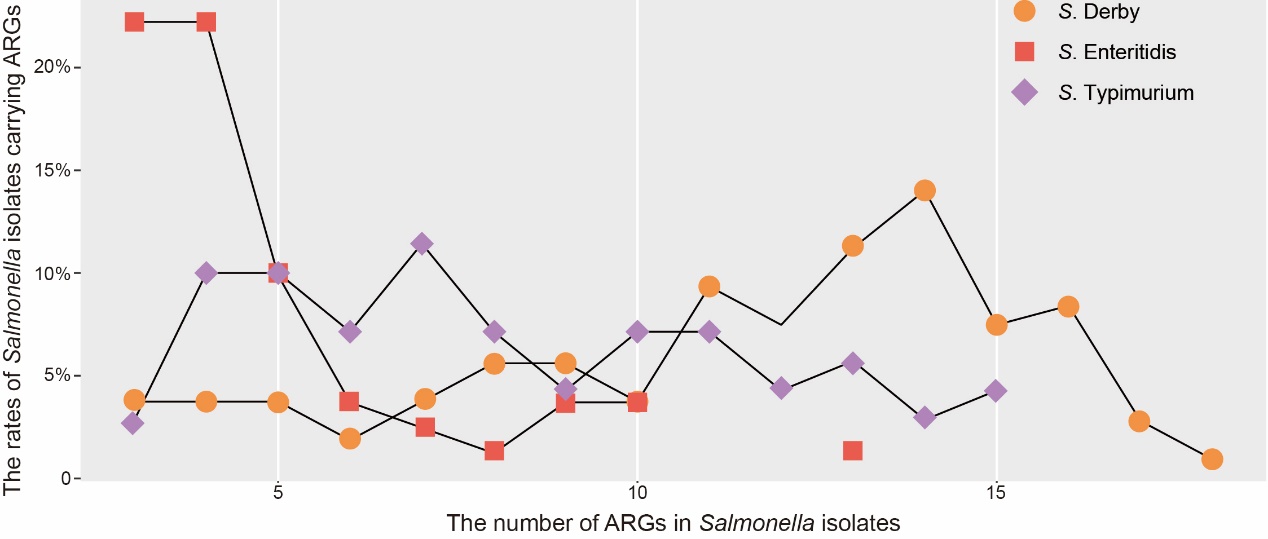


**Figure S7.** The rates of isolates carrying different number of ARGs in *S*. Derby, *S*. Enteridis, and *S*. Typimurium isolates, respectively.


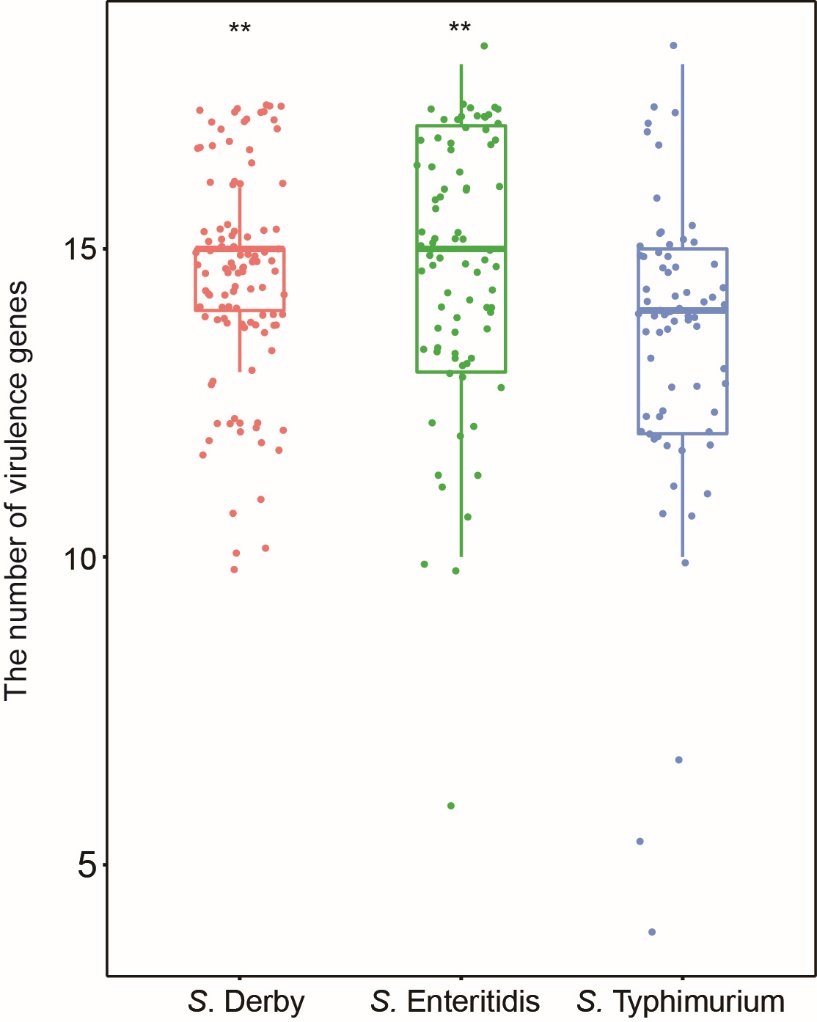


**Figure S8.** Comparison analysis of the number of virulence genes in *S*. Derby, *S*. Enteridis, and *S*. Typimurium isolates, respectively.
